# Intensity Equalization of Bidirectional Fiber Laser Based on a Non-Reciprocal Optical Attenuator

**DOI:** 10.3390/s23094360

**Published:** 2023-04-28

**Authors:** Wenrui Wang, Bowen Xu, Lingyun Ye, Kaichen Song

**Affiliations:** 1 School of Aeronautics and Astronautics, Zhejiang University, Hangzhou 310058, China; wangwenrui@zju.edu.cn (W.W.); kcsong@zju.edu.cn (K.S.); 2 College of Biomedical Engineering & Instrument Science, Zhejiang University, Hangzhou 310058, China; xubw@zju.edu.cn

**Keywords:** bidirectional non-reciprocal optical attenuator, Faraday optical rotation effect, intensity equalization, bidirectional fiber laser

## Abstract

The application of a bidirectional laser requires the laser intensity in both directions to be balanced. However, the CW and CCW light intensities in current bidirectional erbium-doped fiber laser experiments differ due to the gain competition effect. There is no report on equalizing the intensity in the CW and CCW directions. This paper proposes a bidirectional non-reciprocal optical attenuator using the Faraday optical rotation effect. Continuous attenuation adjustment is realized by changing the angle between the polarizer’s transmission axis and the linear polarized light. In this study, we analyzed the influence of different parameters on the device’s performance, built a non-reciprocal attenuator, and tested the bidirectional attenuation curve, which was consistent with the simulation results. The device was integrated into a bidirectional fiber laser, and the light intensity in both directions was balanced through non-reciprocal adjustment. Combined with closed-loop control, the average intensity difference fluctuation between the two directions was controlled at 0.28% relative to the average power, realizing stable long-term bidirectional fiber laser intensity equalization.

## 1. Introduction

In the field of optical detection, the bidirectional output laser has essential application value and broad application scenarios [1,2,3]. There is gain competition in the bidirectional erbium-doped fiber laser: clockwise (CW) and counterclockwise (CCW) lightwaves will compete for the inverted particles, resulting in differences in and continuous fluctuations of CW and CCW light intensity. The difference between the CW and CCW light intensity will expand with increased pump power [4,5,6,7]. The bidirectional output laser requires good coherence between the CW and CCW lasers. The close intensity of the CW and CCW lasers also shows good coherence. Optical signals with close intensities can achieve high optical heterodyne beat efficiency [8,9]. In addition, in the resonant cavity, the difference in light intensity between the CW and CCW lights will cause phase differences due to the Kerr effect, which will affect their resonant frequency and beat frequency [10,11,12]. If the difference between the CW and CCW light intensity changes continuously, a frequency jitter will be introduced into the beat signal, equivalent to introducing noise during frequency detection. This will affect the subsequent application of the bidirectional fiber laser and reduce its potential in other detection fields. If the CW and CCW light intensity can be adjusted in the same optical device at the same time to reduce the difference, it can help to reduce the phase difference between the two lights. At the same time, the beat efficiency of the CW and CCW lights can be increased to improve the overall output power of the system. Therefore, bidirectional fiber lasers with intensity equalization can obtain better detection indicators and have better application prospects.

If the CW and CCW lights can be adjusted differently in the same device simultaneously, a non-reciprocal effect needs to be introduced. The Faraday optical rotation effect is a typical non-reciprocal effect [13,14]. It can be used to make non-reciprocal adjustments in two counter-propagating directions. For example, based on the principle that the Faraday optical rotation angles of different wavelengths are different, isolators with different conduction directions for different wavelengths can be made [15,16,17,18]. Combining the Faraday rotator and quarter-wave plate, phase controllers with different phase delays in different directions can be made [19,20,21], and the Faraday optical rotation effect can also be used to make bidirectional multi-port circulators [22].

With the laser gain unchanged, adjusting the attenuation value of the laser resonator can change the intensity of the laser. Therefore, the intensity of the CW and CCW lasers can be balanced by balancing the attenuation of the CW and CCW resonators. The Faraday rotation effect can change the angle of linearly polarized light, so it is widely used in attenuation regulation applications. Adjustable attenuators and isolators can be made based on the Faraday optical rotation effect [23,24,25,26,27,28,29,30,31]; changing the angle between the linear polarized light and transmission axis of the polarizer realizes a continuous change of attenuation. The existing optical attenuators based on the non-reciprocal Faraday optical rotation effect only focus on the change in optical attenuation in one conduction direction, and there are no reports on adjustable bidirectional non-reciprocal optical attenuators.

Therefore, based on the Faraday optical rotation effect, non-reciprocal attenuation adjustments can be made to CW and CCW resonators by using the angle change between the linear polarized light and the polarizer’s transmission axis, to realize the equalization of the CW and CCW laser intensity of the bidirectional fiber laser, improve the beat efficiency, and reduce other interference caused by the CW and CCW intensity difference. Based on the Faraday optical rotation effect, simultaneous adjustment of CW and CCW resonator attenuation can be realized in the same optical device without adjusting the intensity of the CW and CCW. This can avoid interference introduced by a non-reciprocal optical path, thus simplifying the system design and improving stability.

## 2. Materials and Methods

### 2.1. Intensity Equalization Principle of Bidirectional Fiber Laser

The output intensity of the laser at a stable resonance depends on the gain and attenuation in the resonator. As shown in Equation (1), the saturation intensity I_s_ depends on the nature of the gain material and the frequency of the incident light, small signal gain coefficient g^0^ is independent of the light intensity and depends on the properties of the working material and the pump power, and τ is the attenuation in the resonant cavity [31]. It can be seen from the equation that laser intensity I_m_ can be changed by changing the attenuation in the resonant cavity.

(1) $$I_{m}=\left(g^{0}-\tau \right)I_{s}/\tau$$

In a bidirectional fiber laser, the output light intensity in one direction can be controlled by controlling the attenuation in that direction. If pump power fluctuations and external disturbances are ignored, when the attenuation of the CW resonator is increased, the consumption of reverse particles by the CW light will be reduced, so the CCW light can obtain more reverse particles and gain improved intensity.

Based on the above principle, the intensity of the lights in two directions can be detected, and the attenuation of the CW and CCW resonators can be adjusted according to the intensity difference in the two directions to realize intensity equalization of lasers transmitted in two counter-propagating directions.

### 2.2. Design of Non-Reciprocal Optical Attenuator

Attenuation can be produced by the angle between the linearly polarized light and the polarizer’s transmission axis, and the magnitude of attenuation is related to the angle. At the same time, the polarization rotation direction of linearly polarized light passing through the Faraday rotator is independent of the passing direction; non-reciprocal rotation can be introduced in both forward and backward passing directions through the Faraday rotation effect.

Based on the above two principles, we can design a device to realize non-reciprocal attenuation control of forward and backward directions on the same optical structure.

**Figure 1:**
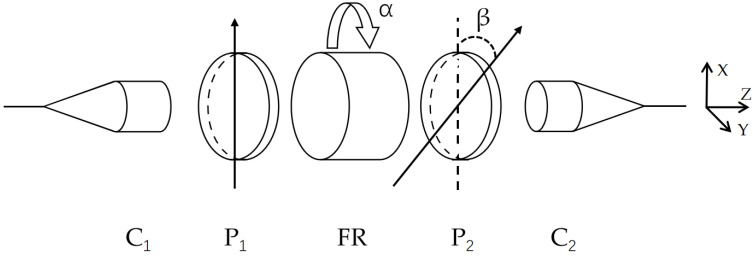
Structure of non-reciprocal optical attenuator, composed of collimator 1 (C_1_), polarizer 1 (P_1_), Faraday rotator (FR), polarizer 2 (P_2_), and collimator 2 (C_2_).

A non-reciprocal optical attenuator mainly comprises two collimators, two polarizers, and a Faraday rotator. The specific structure is shown in Figure 1. Based on the reference coordinate system on the right side of the figure, the transmission axis of polarizer 1 is parallel to the *x*-axis. The rotation direction of the Faraday rotator is from the *x*-axis to the *y*-axis, and the rotation angle is α. The angle between the transmission axis of polarizer 2 and polarizer 1 is β.

The collimator uses a polarization-maintaining (PM) fiber. The polarizer’s transmission axis is aligned with the slow axis of the PM fiber. In the experiment, the Faraday rotation angle is changed by applying magnetic field intensity to the Faraday rotation crystal. The Faraday rotation crystal, polarizer, and collimator used in the experiment all work at 1550 nm.

Assuming the positive direction along the *z*-axis is the forward transmission direction and the negative direction of the *z*-axis is the backward transmission direction, we can analyze the change of polarization angle and attenuation of linearly polarized light in different transmission directions.

**Figure 2:**
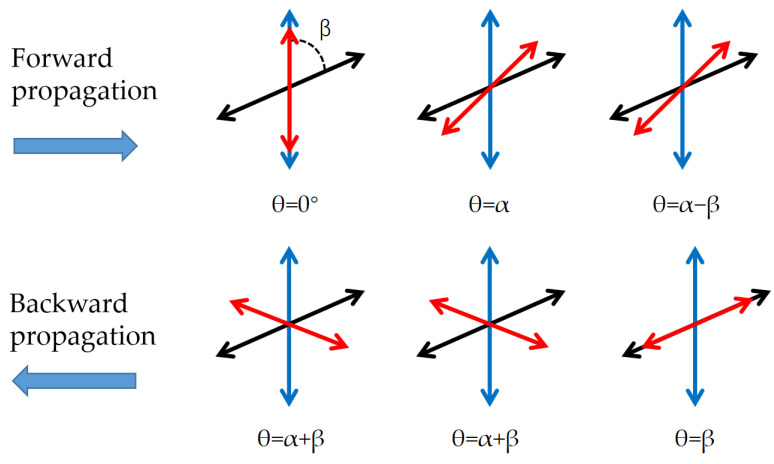
Angle change of linearly polarized light in different propagation directions: forward propagation along +z direction and backward propagation along −z direction. θ is the angle of linearly polarized light.

In Figure 2, the blue two-way arrow indicates the direction of the transmission axis of polarizer 1, the black two-way arrow indicates the direction of the transmission axis of polarizer 2, the angle between the two polarizers is β, and the red two-way arrow indicates the direction of linearly polarized light.

The characteristic after transmission in two directions is calculated through the Jones matrix. The Jones matrix of the linear polarizer is P_1_ and P_2_, and that of the Faraday rotator is FR:

(2) $$\left[P_{1}\right]=\left[\begin{array}{cc} 1 & 0\\ 0 & 0 \end{array}\right]$$

(3) $$\left[P_{2}\right]=\left[\begin{array}{cc} \cos^{2}\beta & \cos\beta \sin\beta \\ \cos\beta \sin\beta & \sin^{2}\beta \end{array}\right]$$

(4) $$\left[\mathrm{FR}\right]=\left[\begin{array}{cc} \cos\alpha & -\sin\alpha \\ \sin\alpha & \cos\alpha \end{array}\right]$$

The Jones matrix in the forward propagation direction is

(5) $$\left[P_{2}\right]\left[\mathrm{FR}\right]\left[P_{1}\right]\left[\begin{array}{c} 1\\ 0 \end{array}\right]=\cos\left(\beta -\alpha \right)\left[\begin{array}{c} \cos\alpha \\ \sin\alpha \end{array}\right]$$

Light intensity after forward propagation I_f_ is

(6) $${I_{f}=\cos}^{2}\left(\beta -\alpha \right)\left(\cos^{2}\alpha +\sin^{2}\alpha \right)=\cos^{2}\left(\beta -\alpha \right)$$

The Jones matrix in the backward propagation direction is

(7) $$\left[P_{1}\right]\left[\mathrm{FR}\right]\left[P_{2}\right]\left[\begin{array}{c} \cos\beta \\ \sin\beta \end{array}\right]=\cos\left(\alpha +\beta \right)\left[\begin{array}{c} 1\\ 0 \end{array}\right]$$

The light intensity after backward propagation I_b_ is

(8) $$I_{b}=\cos^{2}\left(\alpha +\beta \right)$$

By observing the expression of output light intensity in two directions, it can be seen that the Faraday rotator introduces a non-reciprocal rotation of linearly polarized light, which makes the attenuation of forward and backward light different after passing through the linear polarizer. On this basis, forward and backward attenuation can be continuously adjusted by changing the angle of the Faraday rotator. Combined with the light intensity formula, the attenuation characteristic curve of two opposite directions when the Faraday rotation angle changes was simulated and angle β between the two polarizers was assumed to be 45°.

**Figure 3:**
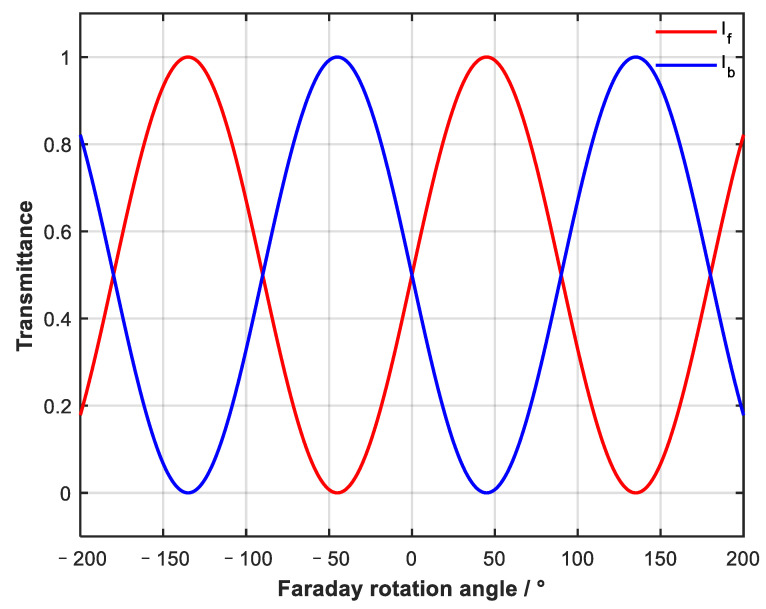
Curve of forward and backward propagation transmittance changing with Faraday rotation angle; angle between polarizer 1 and polarizer 2 is 45°.

As shown in Figure 3, when the angle between the two polarizers is 45°, the adjustment of the Faraday rotation angle to forward and backward attenuation is periodic. The adjustment period of forward and backward attenuation is 180°. The forward and backward attenuation are the same when the Faraday rotation angle is α = k × 90° (k is an integer). When the Faraday rotation angle is α = 45° + k × 180°, the device becomes a forward conduction device. When the Faraday rotation angle is α = −45° + k × 180°, the device becomes a backward conduction device.

The Faraday rotator can realize complete cut-off and conduction in the forward or backward directions within a ±45° rotation range. A continuous adjustment interval exists between complete cut-off and conduction in one direction. Therefore, in the rotation range of ±45°, continuously variable attenuation adjustment can be achieved by continuously changing the Faraday rotation angle.

### 2.3. Analysis of Parameters and Characteristics of Non-Reciprocal Optical Attenuator

In the non-reciprocal optical attenuator, in addition to adjusting the Faraday rotation angle, changing β will also affect the device’s characteristics.

**Figure 4:**
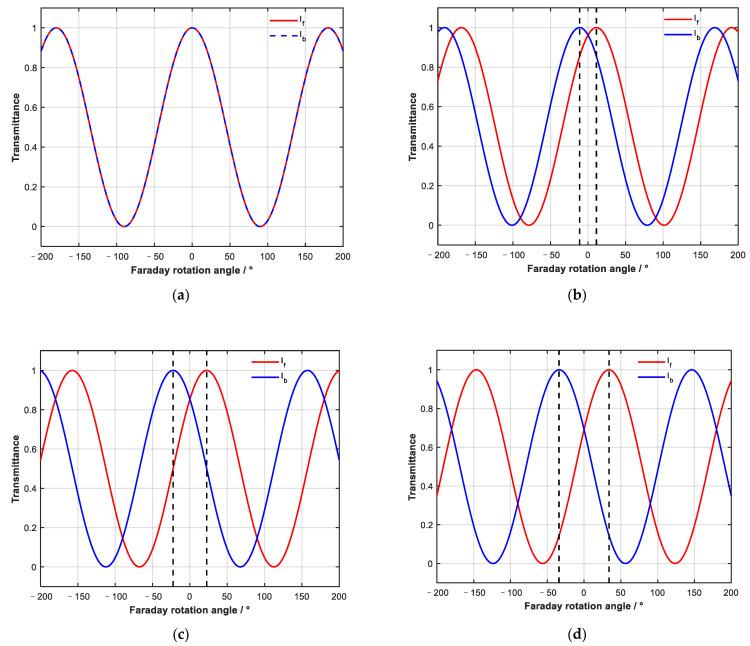
Change of forward and backward propagation transmissivity under different β: (**a**) 0°, (**b**) π/16, (**c**) π/8, and (**d**) 3π/16.

We changed β and observed the influence on the bidirectional adjustment ability of the device. From Figure 4, we can see that regardless of the value of β, forward and backward transmission curves I_f_ and I_b_ show periodic changes; because the period of cos^2^ function is 180°, I_f_ and I_b_ show continuous changes with a period of 180°.

At the same time, when β changes periodically, the device characteristic curve also changes, with a period of 180°. When β changes in the range of 0–180°, the change in the device characteristics shows symmetry, so it is only necessary to study the change in device characteristics with a rotation angle in the range of 0–45°.

It can be seen from Figure 4a that when β is 0°, the forward and backward transmission curves are the same, and the device has no non-reciprocal adjustment function. At the same time, different β values will affect the transmittance when the forward and reverse attenuation are equal. It can be seen from Figure 4 that when β is π/16, the transmittance is about 96% and 4% when the forward and backward attenuation are equal; when β is π/8 the values are 86% and 14%; when β is 3π/16, the values are 69% and 31%; and when β is π/4, the value is 50%.

The value of β also affects the adjustable range of the device, assuming that the maximum non-reciprocal range (within the dotted black lines in Figure 4) is the adjustable range of the device. It can be seen from Figure 4 that when β is small, such as π/16, the adjustable range of the device is 22°; when β is π/8, the value is 45°, and when β is 3π/16, the value is 68°, all of which are less than the 90° range when β is π/4. When the device is an isolator with one-way conduction, if β is not equal to π/4, it cannot be completely cut off in one direction and completely penetrated in the other.

**Figure 5:**
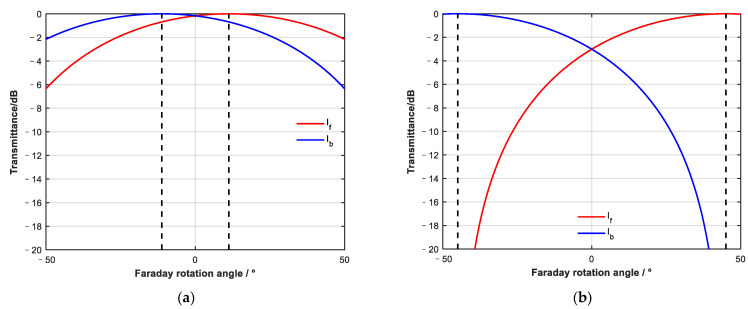
Change of forward and backward propagation transmissivity under different β in logarithmic coordinates: (**a**) π/16 and (**b**) π/4.

It can be seen from Figure 5 that when the logarithmic coordinate system is used to express transmittance, the smaller β is, the smaller the non-reciprocal adjustment angle range is, the smaller the attenuation adjustment range will be, and the smoother the curve will be within the adjustment range.

## 3. Results

### 3.1. Non-Reciprocal Attenuator Test

**Figure 6:**
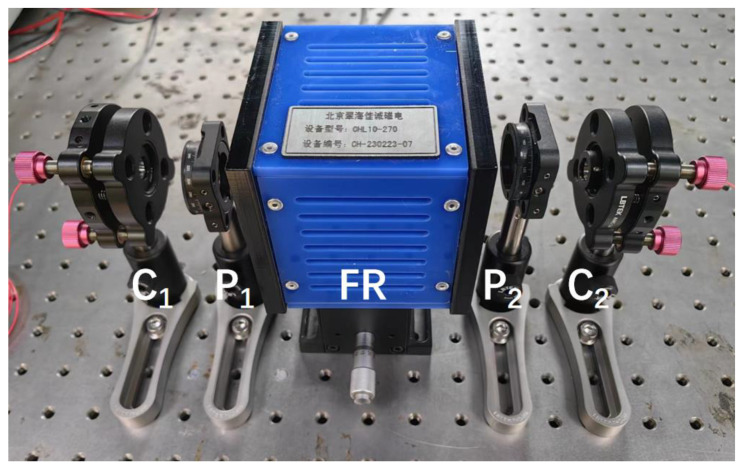
Non-reciprocal optical attenuator, composed of collimator 1 (C_1_), polarizer 1 (P_1_), Faraday rotator (FR), polarizer 2 (P_2_), and collimator 2 (C_2_). The solenoid is made by Beijing Cuihai Jiacheng magnetoelectronics company. Equipment model: CHL10-270; Equipment number: CH-230223-07.

We built a non-reciprocal optical attenuator for testing, and a photo is shown in Figure 6.

**Figure 7:**
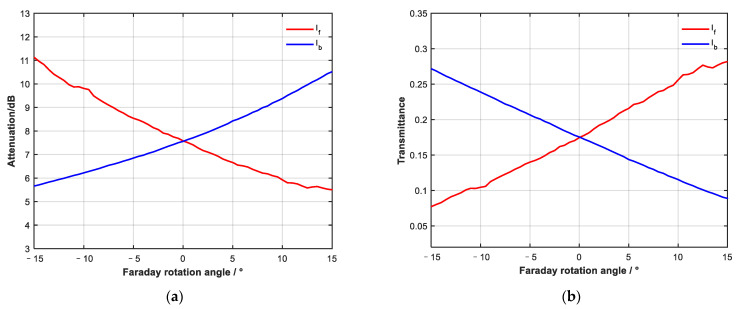
Test result of non-reciprocal optical attenuator: (**a**) in logarithmic coordinates and (**b**) transmittance. This result is consistent with the simulation.

The bidirectional attenuation curve obtained from the test is shown in Figure 7, and β is π/4. When the attenuation in both directions is consistent, the insertion loss of the device is about 7.5 dB. Due to the limitation of magnetic field strength, the adjustment range of the Faraday rotation angle is ±15°. The test results are consistent with the simulation results within the adjustment range. It can be seen from Figure 7a that when the Faraday rotation angle is within ±15°, the adjustment range of attenuation in a single direction can reach 5.5 dB.

### 3.2. Manual Open-Loop Test of Bidirectional Fiber Laser

In addition to testing the adjustable non-reciprocal attenuation characteristics of the attenuator separately, the device was also integrated into the bidirectional fiber laser to observe its effect on output intensity.

**Figure 8:**
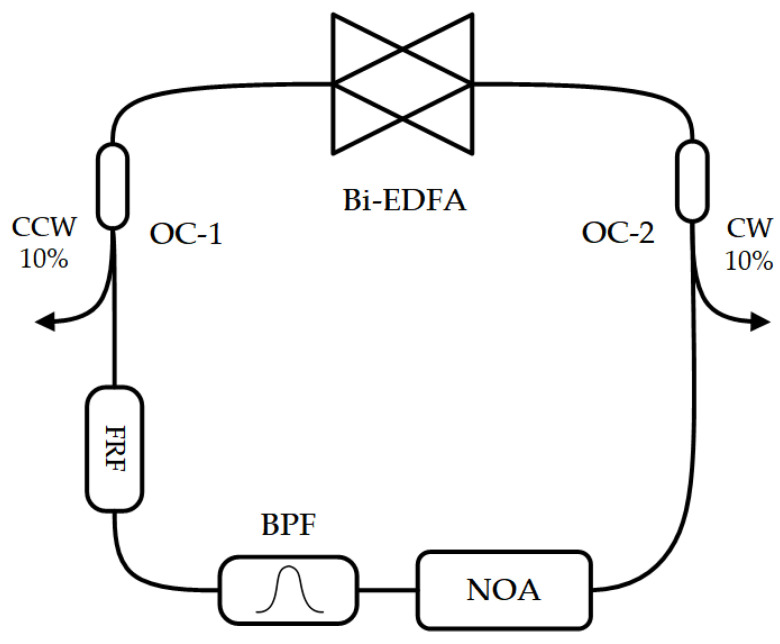
Structure of bidirectional fiber laser, composed of bidirectional EDFA (Bi-EDFA), two 90:10 optical couplers (OC-1, OC-2), a fiber ring filter (FRF), an optical bandpass filter (BPF), and a non-reciprocal optical attenuator (NOA).

The bidirectionally operated erbium-doped fiber amplifier (EDFA) provides the gain in the bidirectional fiber laser. A narrow band-pass filter with a bandwidth of 0.4 nm and a fiber ring filter with a ring length of 60 cm were used to reduce the number of longitudinal modes in the system. The system was a fully polarization-maintaining structure. The monitoring of forward and backward optical power was completed through 90:10 couplers. In Figure 8, the forward transmission direction is clockwise, and the backward transmission direction is counterclockwise.

**Figure 9:**
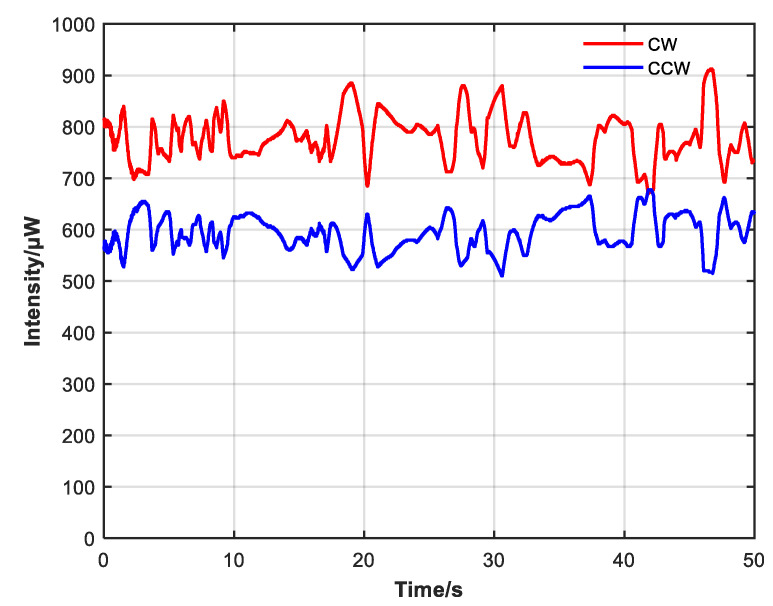
CW and CCW intensity change when non-reciprocal attenuator is not added. Intensity difference between CW and CCW light is large and fluctuates.

Due to the pump and the difference in attenuation of the CW and CCW resonator without the non-reciprocal attenuator, the difference in CW and CCW optical intensity is significant, and there are large intensity fluctuations, as shown in Figure 9. During the observation process, the intensity difference between CW and CCW directions continuously shifted, indicating an unstable state of continuous gain competition. Then, the non-reciprocal optical attenuator was added, and the pump power was adjusted so that the average output intensity of the system was similar before and after adding the attenuator.

**Figure 10:**
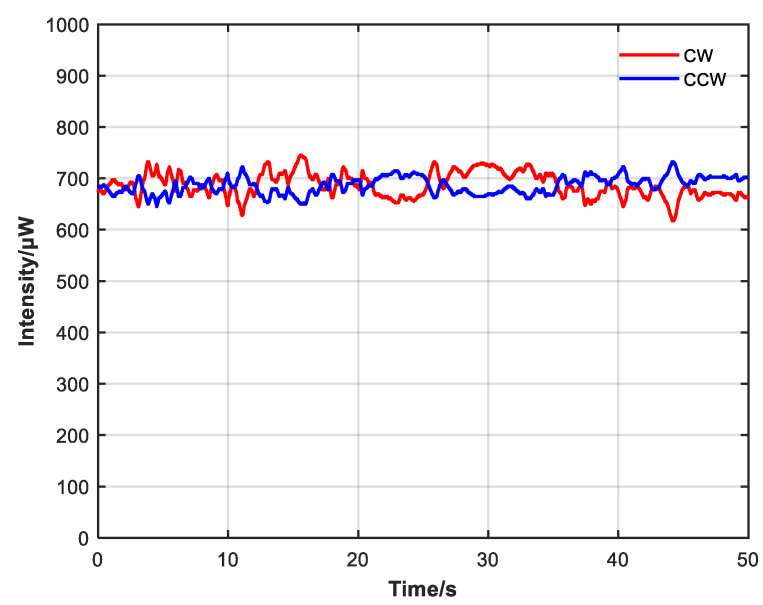
CW and CCW intensity change when non-reciprocal attenuator is added. Intensity difference and power fluctuation are reduced.

Comparing Figure 9 and Figure 10, after adding the non-reciprocal optical attenuator and adjusting so that the CW and CCW intensity were close, the difference between CW and CCW optical intensity and power fluctuation also decreased. Without the non-reciprocal optical attenuator, the average CW intensity was 777.7 μW with a variance of 0.03428, the average CCW intensity was 594.6 μW with a variance of 0.02041, and the average CW and CCW intensity was 686.2 μW. With the non-reciprocal optical attenuator, the average CW intensity was 687.2 μW with a variance of 0.00891, the average CCW intensity was 686.4 μW with a variance of 0.00430, and the average CW and CCW intensity was 686.8 μW.

**Figure 11:**
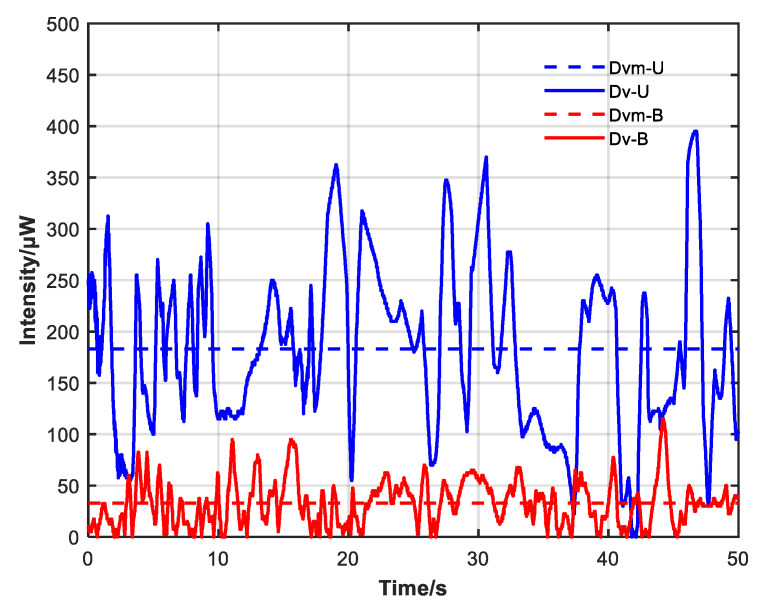
Change curve of difference between CW and CCW light intensity with and without non-reciprocal attenuator. Dv-U: difference in light intensity without non-reciprocal attenuator. Dvm-U: average of Dv-U. Dv-B: difference in light intensity with non-reciprocal attenuator. Dvm-B: average of Dv-B.

Figure 11 shows the CW and CCW intensity difference before and after adding the non-reciprocal optical attenuator; blue indicates the condition without the attenuator, red indicates the condition with the attenuator added and adjusted close to the power position, and the dotted line indicates the average value of the difference between the two conditions.

The average CW and CCW intensity difference before adding the non-reciprocal optical attenuator was 183.2 μW relative to an average power ratio of 26.7%, with a variance of 0.10417. After adding the non-reciprocal optical attenuator, the average value decreased to 32.9 μW relative to an average power ratio of 4.8%, with a variance of 0.00749. The peak CW and CCW intensity difference also decreased from 395 to 115 μW after the non-reciprocal optical attenuator was added.

Manual adjustment can suppress the difference between CW and CCW laser intensity to a certain extent. Through comparison, it can be seen that the manual open-loop adjustment is inaccurate; there is still a large difference between the CW and CCW intensity, and the significant intensity fluctuation cannot be suppressed. Moreover, the intensity difference between the two directions will gradually expand when external conditions change over time, so better adjustment logic needs to be considered.

### 3.3. Closed-Loop Test of Bidirectional Fiber Laser

**Figure 12:**
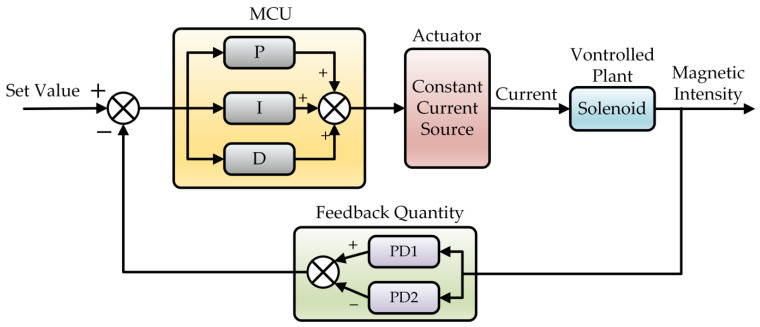
Block diagram of closed-loop control system.

In order to overcome the defects of open-loop control, a closed-loop control system was built (Figure 12) to detect the difference between CW and CCW intensity using two 80 MHz photoelectric detectors (PDs) and calculate the control quantity according to the difference and PID algorithm in MCU, with a control cycle of 1 ms. Finally, the solenoid current was controlled to change the Faraday rotator’s rotation angle, and then different attenuation values were introduced to the CW and CCW directions to achieve the purpose of intensity equalization.

**Figure 13:**
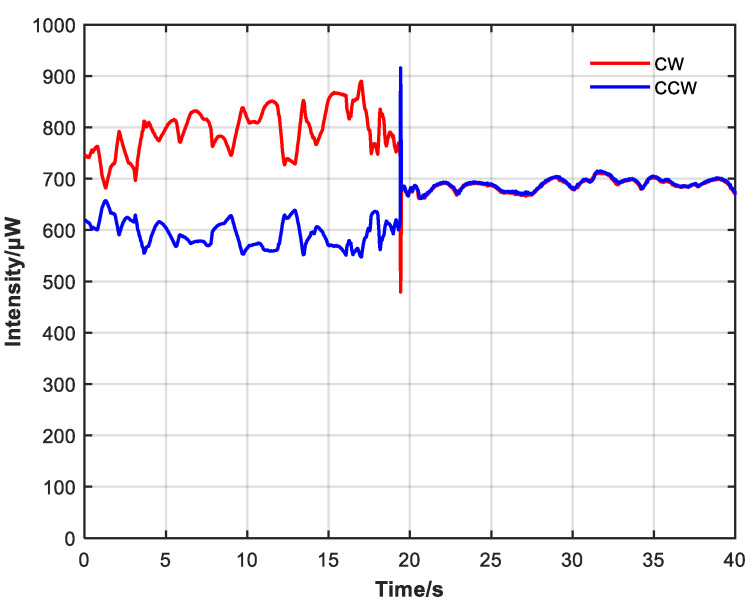
CW and CCW intensity change before and after start of closed-loop control; effect of closed-loop control is obvious.

As shown in Figure 13, the control effect is evident after the closed-loop control is started, and the difference between CW and CCW intensity is well suppressed. During the observation, the intensity difference between CW and CCW directions remained constant and did not drift over time.

**Figure 14:**
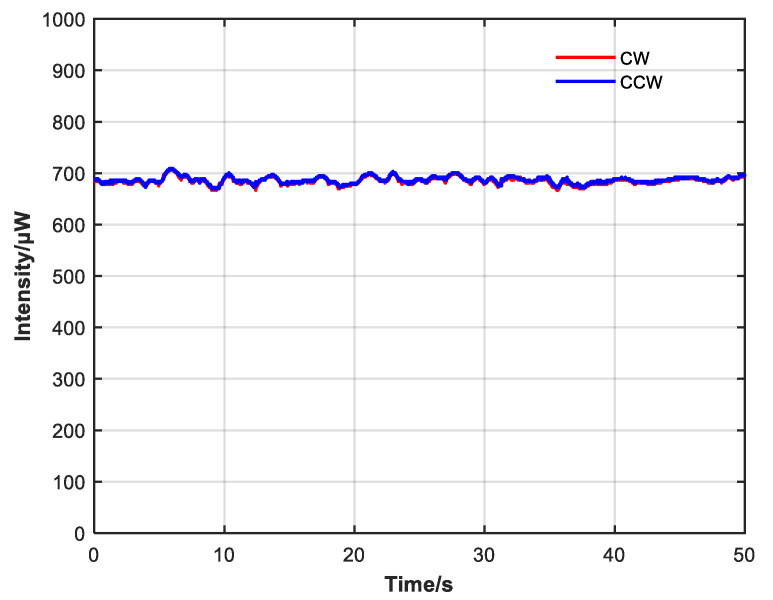
CW and CCW intensity changes within 50 s after adding closed-loop control.

**Figure 15:**
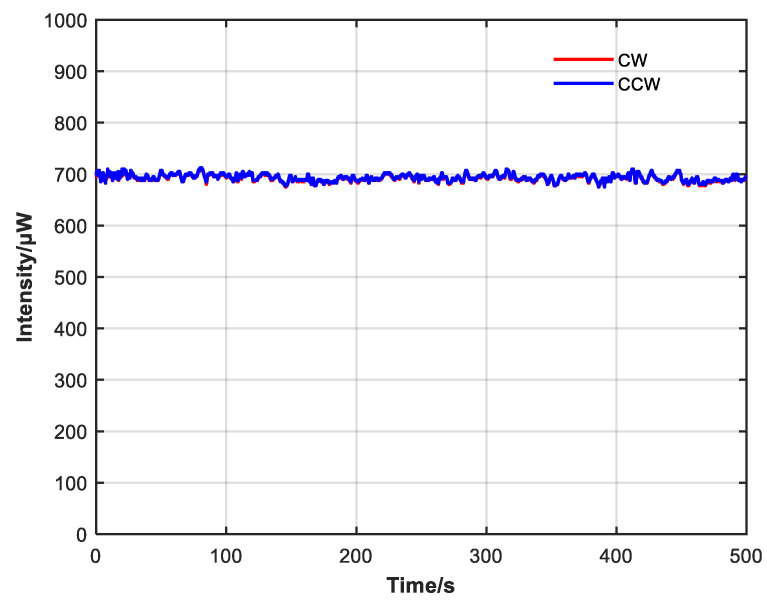
CW and CCW intensity changes within 500 s after adding closed-loop control.

It can be seen from Figure 14 and Figure 15 that CW and CCW intensity are very close at 50 and 500 s, which suppresses the power fluctuation with large amplitude and the expansion of the power difference caused by the long operation time of the system and achieves a good closed-loop control effect.

**Figure 16:**
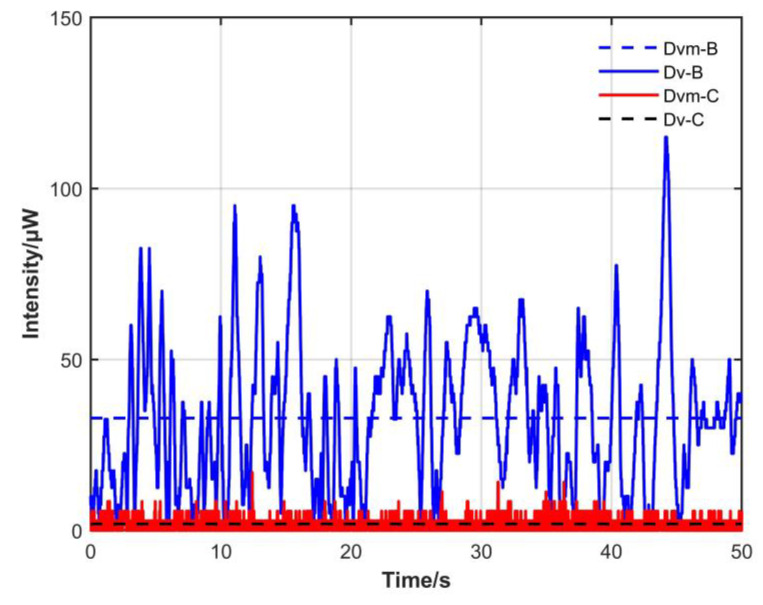
Change curve of difference between CW and CCW light intensity with and without closed-loop control. Dv-B: difference in light intensity without closed-loop control. Dvm-B: average of Dv-B. Dv-C: difference in light intensity with closed-loop control. Dvm-C: average of Dv-C.

The average CW intensity after introducing closed-loop control was 685.1 μW, with a variance of 0.00054. The average CCW intensity was 686.6 μW, with a variance of 0.00051. The average CW and CCW intensity was 685.9 μW. It can be seen from Table 1 that average CW and CCW intensity difference before introducing closed-loop control was 32.9 μW relative to an average power ratio of 4.8%, with a variance of 0.00749. After introducing closed-loop control, the average difference decreased to 1.89 μW relative to an average power ratio of 0.28%, with a variance of 0.00002. The peak value of CW and CCW intensity difference also decreased from 115 to 17 μW after introducing closed-loop control. It can be seen from Figure 16 that after adding closed-loop control, not only the average difference decreased significantly, but also the variance decreased significantly. Closed-loop control can suppress differences as well as improve the stability of optical power.

## 4. Conclusions

In this paper, a bidirectional non-reciprocal optical attenuator was designed based on the Faraday optical rotation effect, and the influence of various parameters of the device on the overall performance was calculated, guiding subsequent experiments and design. The device was built and tested according to the simulation results, and its characteristic curve is consistent with the simulation results. The device was integrated into the bidirectional fiber laser to verify the balance effect of the bidirectional non-reciprocal optical attenuator on the output intensity of the laser through experimentation. In the case of manual open-loop control, the average bidirectional optical intensity difference decreased by about 5.6 times, the ratio of average intensity decreased by about 5.6 times, and the variance decreased by about 13.9 times. On this basis, closed-loop control was introduced. Compared with manual open-loop control, the average bidirectional light intensity difference decreased by about 17.4 times, the ratio of average intensity decreased by about 17.2 times, and the variance decreased by about 374.5 times. The non-reciprocal optical attenuator realized non-reciprocal attenuation control of CW and CCW light simultaneously on the same optical path structure. Combined with closed-loop control, it can achieve a stable, long-term bidirectional power balance, which is conducive to the follow-up application of bidirectional fiber lasers.

## Figures and Tables

**Table 1:** Comparison of results under various conditions.

Condition	Average Intensity (μW)	Average Intensity Difference (μW)	Percentage of Intensity Difference	Variance of Difference
Non-reciprocal attenuator is not added	686.2	183.2	26.7%	0.10417
Closed-loop control is not introduced	686.8	32.9	4.8%	0.00749
Closed-loop control is introduced	685.9	1.89	0.28%	0.00002

## Data Availability

The data that support the findings of this study are available from the corresponding author upon reasonable request.
